# Quasi-experimental evaluation of municipal ice cleat distribution programmes for older adults in Sweden

**DOI:** 10.1136/ip-2022-044808

**Published:** 2023-05-22

**Authors:** Elin Eklund, Robin Holmberg, Mikael Svensson, Johanna Gustavsson, Carl Bonander

**Affiliations:** 1 School of Public Health and Community Medicine, University of Gothenburg, Gothenburg, Sweden; 2 Department of Political, Historical and Cultural Studies, Karlstad University, Karlstad, Sweden; 3 Centre for Societal Risk Research, Karlstad University, Karlstad, Sweden; 4 Department of Pharmaceutical Outcomes and Policy, University of Florida, Gainesville, Florida, USA

**Keywords:** Fall, Community, Older People, Equipment

## Abstract

**Introduction:**

Fall injuries caused by icy road conditions are a prevalent public health problem during winters in Sweden, especially in older populations. To combat this problem, many Swedish municipalities have distributed ice cleats to older adults. While previous research has shown promising results, there is a lack of comprehensive empirical data on the effectiveness of ice cleat distribution. We address this gap by investigating the impact of these distribution programmes on ice-related fall injuries among older adults.

**Methods:**

We combined survey data on ice cleat distribution in Swedish municipalities with injury data from the Swedish National Patient Register (NPR). The survey was used to identify municipalities that have distributed ice cleats to older adults at some point between 2001 and 2019. Data from NPR were used to identify municipality-level data on patients who have been treated for injuries related to snow and ice. We used a triple differences design—a generalisation of difference in differences—that compared ice-related fall injury rates before and after intervention in 73 treatment and 200 control municipalities, with unexposed age groups serving as within-municipality controls.

**Results:**

We estimate that the average ice cleat distribution programmes reduced ice-related fall injury rates by −0.24 (95% CI −0.49 to 0.02) per 1000 person-winters. The impact estimate was larger in municipalities that distributed more ice cleats (−0.38 (95% CI −0.76 to –0.09)). No similar patterns were found for fall injuries unrelated to snow and ice.

**Conclusion:**

Our results suggest that ice cleat distribution can decrease the incidence of ice-related injuries among older adults.

**WHAT IS ALREADY KNOWN ON THIS TOPIC**

- Ice cleat distribution may be a cost-effective way to reduce the burden of ice-related fall injuries, but comprehensive impact evaluations are lacking.

**WHAT THIS STUDY ADDS**

- We perform a comprehensive, quasi-experimental evaluation of 73 municipal ice cleat distribution programmes targeting older adults in Sweden. Our primary estimate suggests that ice-related injury incidence was 8.2% lower on average after ice cleat distribution.

**HOW THIS STUDY MIGHT AFFECT RESEARCH, PRACTICE OR POLICY**

- Our study reaffirms previous evidence suggesting that distributing ice cleats to older adults may be an effective prevention measure in settings affected by snow and ice.

## Introduction

Fall injuries that occur outdoors are often associated with environmental risk factors such as snow and ice, which are very common during winter in Nordic countries. As a result, ice-related fall injuries are a prevalent public health problem in Sweden.^1^ In previous studies, fall injuries have been found to be associated with weather conditions in interaction with individual characteristics such as high age.^1^ The risk of being injured in an ice-related fall increases with age,^2^ which implies a need for interventions targeting older adults. Research suggests that ice cleats can reduce the risk of ice-related fall injuries.^2–5^ Distributing ice cleats could, therefore, potentially complement other community interventions, such as clearing snow from roads and walkways.^6 7^

Over the past decade, about 25% of Sweden’s 290 municipalities have distributed and offered ice cleats to older citizens to help combat the seasonal rise in ice-related fall injuries that typically occur during the Swedish winters. Previous research suggests that exposure to these programmes is associated with greater ice cleat use among older adults, especially in municipalities with high distribution rates per citizen.^8^ Model-based economic evaluations have also found that ice cleat distribution is likely to be cost-effective.^9 10^ However, there is still a lack of comprehensive evidence on how the distribution of ice cleats impacts fall injury rates. To our knowledge, only one study has directly investigated changes in fall-related injury rates following an ice cleat distribution programme, and the estimates from this study are limited to a single city (Gothenburg, Sweden).^7^ While their results showed a short-term reduction, the programme in Gothenburg was also quite successful in reaching its target population (62% of all eligible citizens collected a pair of ice cleats^7^). Meanwhile, process evaluation data indicate that municipalities have variations in programme designs, which can impact programme effectiveness in terms of reach.^6^ It, therefore, remains unclear whether these programmes have had an impact on ice-related fall injuries. While greater distribution rates seem to lead to larger increases in ice cleat use,^8^ it also remains unclear if these results translate to greater impacts on ice-related injury rates in municipalities with greater reach. In this study, we aimed to address these issues by conducting a comprehensive impact evaluation of the ice cleat distribution programmes on ice-related injury rates among older adults in Swedish municipalities.

## Methods and materials

### Data collection

#### Intervention data

In June 2019, we sent an electronic survey to all municipalities in Sweden (n=290) to collect data on ice cleats distribution programmes, with non-responding municipalities receiving up to 4 reminders (the final reminder was sent in October 2019). In the survey, we asked if the municipality had ever distributed ice cleats. If they answered yes, we collected data on implementation dates, targeted age groups, programme costs and how many ice cleats they distributed. Further details about the intervention data collection can be found in Holmberg *et al*.^6^

#### Injury outcome data

We used municipality-level data from the Swedish National Patient Register (NPR)^11^ to estimate the number of patients treated in inpatient care or at hospital-based outpatient physician visits for injuries related to snow and ice during the study period 2001–2019. Per our request, the National Board of Health and Welfare provided aggregated data on the number of patients with International Classification of Diseases, 10th revision (ICD-10) external cause code W00 (Fall due to ice and snow) stratified by municipality, year, month and age. To avoid double counting (eg, due to readmission), they only counted the same patient once per calendar year for the same diagnosis. To calculate rates per 1000 population, we combined these patient numbers with population data from Statistics Sweden.^12^

To assess the risk of bias, we also collected corresponding data on the number of patients with external cause codes W01–W18 (Falls due to other specified causes unrelated to snow and ice) as a negative control outcome.^13^ We refrained from collecting mortality data because deaths due to falls on snow and ice are very uncommon in Sweden.^9^

### Study design

We used a triple differences design^14^ to estimate the average impact of the ice cleat distribution programmes. Like a conventional difference-in-differences approach,^15^ the design controls for any time-invariant confounders by using preintervention data among the ages eligible for ice cleat distribution (‘eligible ages’), as well as national time trends by using concurrent outcome data from municipalities without ice cleat programmes (“control municipalities”). Our triple differences design also includes an internal control group consisting of within-municipality age groups that were ineligible for ice cleat distribution (‘ineligible ages’ defined here as 1–15 years younger than the age of eligibility), which allowed us to control for local time trends (eg, weather shocks and concurrent interventions). In control municipalities, we defined 65+ years as the eligible age group and 50–64 years as internal controls, as this is the most common age of eligibility for ice cleat distribution used by Swedish municipalities.^6^

### Statistical analysis

For the statistical analysis, we constructed a panel dataset stratified by municipality, time, and eligible and ineligible age groups. We defined time intervals in winter periods (eg, 2003/2004) by aggregating data from 1 October to 30 April, as these months approximately capture the period at risk for ice-related injuries in Sweden (see online supplemental figure S1). The study period spans from the winter of 2001/2002 to 2018/2019 (18 winters). If a municipality implemented a programme during a given winter, eligible ages within that municipality were coded as treated from that period until the end of the study, reflecting the possibility that behavioural responses may persist even after the distribution has ended.

10.1136/ip-2022-044808.supp1 Supplementary data

**Figure 1:**
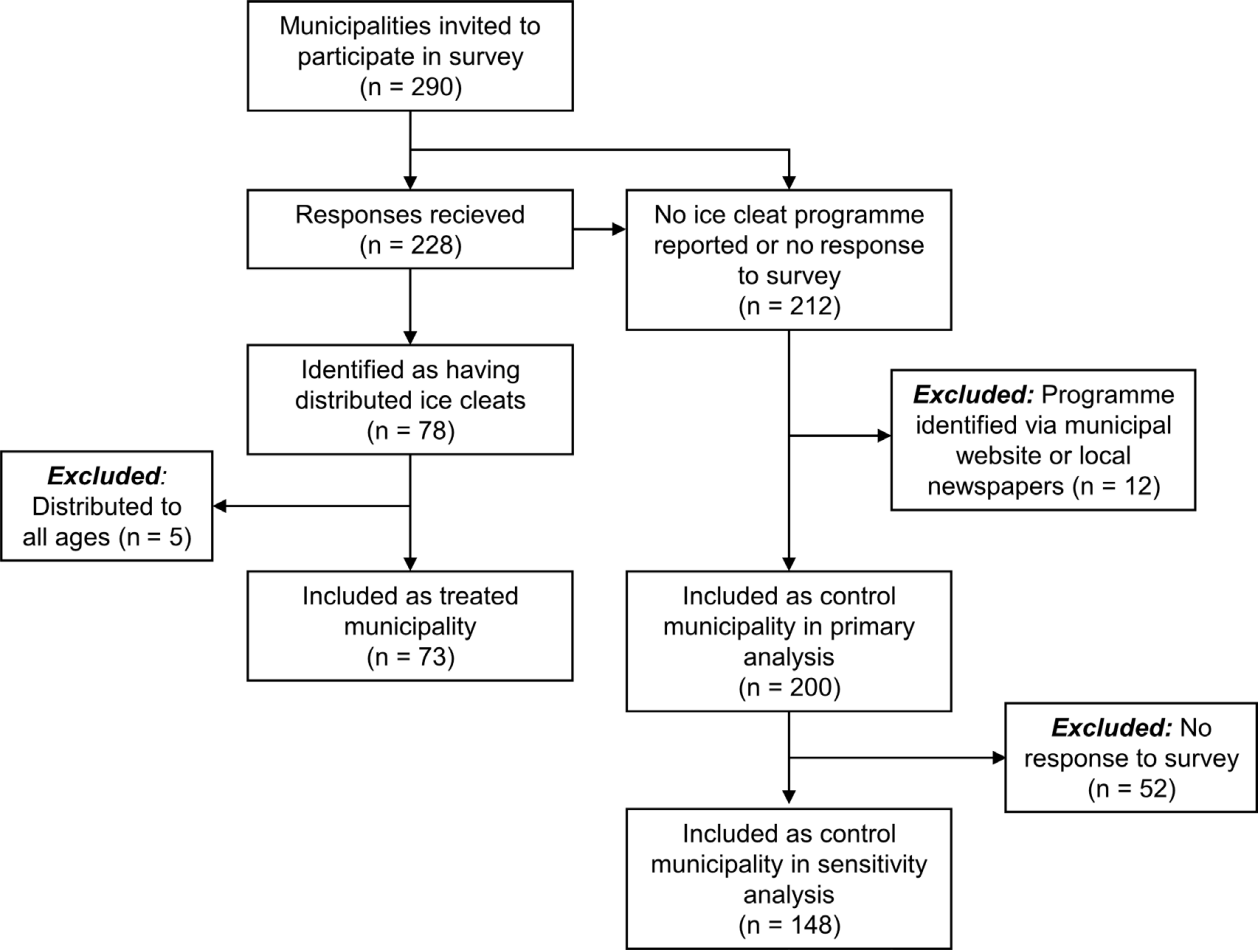
Flow chart of the study sample selection process.

The programmes were implemented in different years (typically referred to as staggered adoption). It was recently discovered that two-way fixed effects models—the models typically applied in difference-in-differences studies—may be biased with this data structure.^16^ To estimate the impact of ice cleat distribution, we, therefore, applied an alternative imputation approach proposed by Borusyak *et al*,^17^ which does not suffer from bias due to staggered adoption.

The imputation-based method estimates the impact by first fitting a fixed effects regression model to not-yet-treated observations (ie, observations from the preperiod or unexposed groups). It then uses the estimated model to impute the expected counterfactual postperiod injury rates in all programme municipalities. It then calculates winter-and-municipality-specific impact estimates by taking the difference between the observed injury rates and the imputed counterfactual rates, and finally averages the estimates across programme municipalities and postintervention time points to estimate average effects. We performed the analysis in Stata V.17 (StataCorp), using the DID_IMPUTATION module,^18^ which performs the imputation and also accounts for within-municipality autocorrelation using cluster-robust standard errors. For further details, see online supplemental materials.

In our primary analysis, we aimed to estimate the average intention-to-treat effect^19^ of the programmes, which reflects the effectiveness of the programmes under ‘real-world’ conditions (including limited reach and adherence). In a secondary analysis, we also estimated the efficacy in a scenario where all targeted citizens collect a pair of ice cleats (ie, when the reach is perfect). To do this, we divided the municipality-specific impact estimates by municipality-specific reach before estimating the average programme impact, as proposed by Borusyak *et al*.^17^ Following Holmberg *et al*,^6 8^ we defined reach as the number of ice cleats distributed divided by the size of the postintervention target population in each programme municipality.

### Sensitivity analyses

The validity of difference-in-differences analyses relies on the parallel trend assumption, which, in essence, means that the groups must have followed the same trend on the outcome in a counterfactual scenario without ice cleat programmes.^20^ To probe this assumption, we checked for differential pretrends visually. We also performed an F-test on time-specific placebo estimates up to 10 years before the implementation winter to assess if pre-existing differences jointly differed from zero, which if true would imply that any observed intervention impacts started occurring even before the intervention started (eg, due to non-parallel trends or anticipation effects).^17^ To further assess the risk of bias due to non-parallel trends, we performed a synthetic control analysis^21^ using the Bayesian dynamic multilevel latent factor modelling approach proposed by Pang *et al*,^22^ which relaxes the parallel trend assumption by modelling deviations from the national common trend using latent factors (see online supplemental materials for details). Finally, we conducted a falsification test by using fall injuries unrelated to snow and ice (ICD-10 codes W01–W18) as a negative control outcome.^13^

### Patient and public involvement

No patients or members of the public were involved in the design of the study.

## Results

We received a response from 228 (78.6%) out of the 290 municipalities invited to answer our survey (figure 1). Of these, 78 municipalities responded that they had distributed ice cleats. Five of these were excluded as they reported having distributed ice cleats to all ages, and therefore, cannot be analysed using our triple difference methodology. All remaining municipalities—that is, those who answered that they had not distributed ice cleats or did not respond to our survey—were assessed for eligibility to be included as controls. To validate the survey responses, we searched online for communications about ice cleat distribution programmes for all 290 Swedish municipalities (information was usually available on municipal websites or reported in local newspapers). This procedure identified 12 additional municipalities with distribution programmes. Two of these had participated in our survey but reported having no programme. Due to the inconsistency and lack of programme data, these 12 were all excluded from the study. The final study sample included 273 municipalities (73 with intervention, 200 controls; figure 1). As a sensitivity analysis, we also restricted the controls to those who responded to the survey (n=148).

### Descriptive programme data

Table 1 contains descriptive data on the 73 included ice cleat programmes. The majority (84.9%) of programmes had set the age of eligibility for ice cleat distribution to 65+ years. Most programmes (78.1%) were implemented late in the study period (between 2015 and 2019), with a mean observation time of 14.5 winters before and 3.5 winters after intervention (see online supplemental figure S2 for exact data on implementation period per municipality). The programmes varied greatly in terms of reach, with a mean number of ice cleat pairs distributed per eligible citizen at 0.40 (min: 0.01, max: 1.08). This number was highly correlated with the number of ice cleats purchased (Spearman’s r=0.89, p<0.001; mean: 0.48 pairs per eligible citizen). Combining the cost data provided by the municipalities with population numbers, we estimate that the mean programme cost per eligible citizen was €3.069 in 2018 (table 1).

**Table 1:** Characteristics of ice cleat distribution programmes for older adults included in the study (n=73)

Characteristic	Descriptive data	n missing
Programme municipalities—n	73	
Ages eligible for ice cleat distribution—n (%)		0
65+ years	62 (84.9)	
70+ years	7 (9.6)	
75+ years	3 (4.1)	
80+ years	1 (1.4)	
Implementation period—n (%)		0
Between 2005 and 2009	4 (5.5)	
Between 2010 and 2014	12 (16.4)	
Between 2015 and 2019	57 (78.1)	
Observation time—mean (min-max)		0
Before distribution	14.5 winters (4–17)	
After distribution	3.5 winters (1–14)	
Reach*—mean (min–max)	0.40 (0.01–1.08)	7 (9.6%)
Purchased ice cleat pairs per eligible citizen—mean (min–max)	0.48 (0.02–1.34)	23 (31.5%)
Programme cost per eligible citizen, 2018 Euros—mean (min–max)	€3.069 (0.039–15.861)	11 (15.1%)

*Reach is defined as the number of distributed ice cleats per eligible citizen. A number below 1 indicates that less than one ice cleat pair was distributed per citizen, and a number above 1 indicates that more than one pair was distributed per eligible citizen.

All 73 municipalities provided the essential programme data required for our intention-to-treat analysis (table 1). Seven municipalities did not provide data on the number of distributed ice cleats and were therefore excluded from the (secondary) efficacy analysis.

### Descriptive injury data

Our analysis is based on data from 132 465 patients treated for injuries due to an ice-related fall (numbers by age group and intervention status are presented in table 2). Considering the entire study period, the mean incidence of ice-related falls in the eligible age groups was 2.84 per 1000 person-winters in control municipalities and 2.39 per 1000 person-winters in programme municipalities. The mean incidence increased over time in all groups (online supplemental figure S3), but the increase was smaller in eligible than ineligible ages within programme municipalities (table 2). Corresponding data on the negative control outcome can be found in table 2 and online supplemental figure S4.

**Table 2:** Descriptive injury data by intervention, period and age groups for fall injuries related to snow and ice (primary outcome) and for other specified injuries unrelated to snow and ice (negative control outcome)

Group and period	Snow and ice-related fall injuries (ICD-10: W00)		Other specified fall injuries unrelated to snow and ice (ICD-10: W01–W18)	
	No of injury patients	Mean incidence per 1000 person-winters (min–max)	No of injury patients	Mean incidence per 1000 person-winters (min–max)
Control municipalities (all periods)				
Eligible ages	49 963	2.84 (0–15.95)	390 509	20.72 (0–39.92)
Ineligible ages	45 923	2.67 (0–17.42)	158 187	8.60 (0–35.01)
Programme municipalities (all periods)				
Eligible ages	18 193	2.39 (0–11.04)	188 323	21.65 (4.50–75.97)
Ineligible ages	18 386	2.29 (0–11.80)	80 930	8.72 (0.66–21.61)
Programme municipalities (preperiod)				
Eligible ages	13 583	2.33 (0–11.04)	137 545	21.21 (4.5–63.81)
Ineligible ages	13 910	2.18 (0–10.53)	58 576	8.36 (0.66–20.13)
Programme municipalities (postperiod)				
Eligible ages	4610	2.67 (0–10.84)	50 778	23.55 (8.98–75.97)
Ineligible ages	4476	2.76 (0–11.80)	22 354	10.25 (1.73–21.61)

Notes: Programme municipalities (n=73) are municipalities that have distributed ice cleats; control municipalities are all other municipalities in the study sample (n=200). All periods reflect the entire study period from the winter of 2001/2002 to the winter of 2018/2019. Preperiod is the period before intervention in programme municipalities and postperiod is the period after intervention in programme municipalities (not applicable for control municipalities). Eligible ages are defined as all ages above the age of eligibility in programme municipalities, and ineligible ages are 1–15 years younger than the age of eligibility. In control municipalities, the eligible ages are defined as 65+ years and ineligible ages as 50–64 years, reflecting the most common age ranges in programme municipalities.

ICD-10, International Classification of Diseases, 10th revision.

### Estimated impact of ice cleat distribution

The results from the triple differences analysis are presented in table 3. The primary analysis suggests an average intention-to-treat effect of −0.24 (95% CI −0.49 to 0.02) ice-related fall injuries per 1000 person-winters, which corresponds to a −8.2% change. Scaling the estimates by municipality-specific reach implies that the impact under ideal conditions is −0.38 (95% CI −0.76 to –0.09) ice-related fall injuries per 1000 person-winters, which corresponds to a −12.5% change.

**Table 3:** Estimated average impact of ice cleat distribution programmes in 73 Swedish municipalities on snow and ice-related fall injuries among older adults (primary analysis) and fall injuries unrelated to snow and ice (negative control analysis)

Model and estimate	Estimated impact per 1000 person-winters (95% CI)	Impact in relative terms	P value for impact	P value from parallel pre-trends test*
Primary analysis				0.143
Intention to treat†	−0.24 (−0.49, 0.02)	−8.2%	0.066	
Efficacy‡	−0.38 (−0.76, −0.09)	−12.5%	<0.001	
Negative control analysis§				0.096
Intention to treat†	0.06 (−0.76, 0.79)	0.3%	0.881	
Efficacy‡	0.02 (−0.62, 0.65)	0.1%	0.995	
Synthetic control analysis¶				N/A
Intention to treat†	−0.22 (−0.44, 0.00)	−7.6%	0.055	
Survey respondents only**				0.169
Intention to treat†	−0.27 (−0.53, −0.01)	−9.2%	0.043	
Efficacy‡	−0.42 (−0.72, −0.11)	−13.6%	0.006	

*A joint significance test (F-test) on time-specific preintervention impact estimates up to 10 winters before the intervention takes place. A significant test suggests a risk of bias. Not estimable in the synthetic control analysis.

†Average effectiveness estimate using a binary intervention variable. The estimate reflects the average expected impact under typical conditions. Based on data from all 73 programme municipalities.

‡Average efficacy estimate estimated by scaling municipality-specific impact estimates by municipality-specific reach (ie, the number of ice cleat pairs distributed per eligible citizen). The estimate reflects the expected impact when one ice cleat pair distributed per citizen. For reference, the average programme municipality distributed 0.4 ice cleat pairs per eligible citizen (table 1). This estimate is based on data from the 66 programme municipalities within non-missing distribution data.

§The negative control outcome is the incidence of fall injuries unrelated to snow and ice per 1000 population (ICD-10 codes W01-W18), which should not be affected by ice cleat distribution. A significant impact suggests a risk of bias.

¶Results from an analysis that is more robust potential violations of the parallel trend assumption (see online supplemental file 1 for details). Efficacy could not be estimated in this analysis.

**Results from an analysis that restricts the control group to non-programme municipalities that responded to our survey (n controls=148).

ICD, International Classification of Diseases, 10th revision; N/A, not available.

#### Negative control analysis

The negative control analysis showed no evidence of effects on injuries unrelated to snow and ice (table 2).

#### Pretrends assessment

There were no visual signs of pretrends (online supplemental figure S5) and the pretrends tests did not identify significant ‘effects’ before the start of the interventions (table 2).

#### Sensitivity analyses

The Bayesian synthetic control analysis, which is more robust to deviations from the parallel trend assumption, produced results that were similar to the primary analysis (table 2; see online supplemental files for detailed results). Restricting the control sample to municipalities that responded to our survey also had limited influence on the results (table 2).

## Discussion

This study aimed to investigate the average impact of Swedish municipal ice cleat distribution programmes on ice-related fall injuries among older adults. Using a quasi-experimental design, we found evidence suggesting that distributing ice cleats may reduce injury rates by about 8% with a mean of 3.5 years of follow-up in the average programme municipality and by 12.5% if one ice cleat pair is distributed per eligible citizen.

To our knowledge, this is the first comprehensive impact evaluation investigating injury outcomes following multiple ice cleat distribution programmes. Overall, our findings are consistent with previous research. In terms of injury impacts, Bonander and Holmberg^7^ also found evidence of a reduction in emergency department visits for ice-related falls following a distribution programme in Gothenburg, Sweden. We have also found an association between ice cleat distribution and increased ice cleat use among older adults living in municipalities with ice cleat programmes,^8^ and data from other studies suggest that using ice cleats can reduce the risk of ice-related injuries.^3–5^ It, therefore, appears plausible that the reductions we observed in this study are caused by increases in ice cleat use. In fact, a population impact analysis using external data on estimated increases in ice cleat use^8^ and data on the effects of ice cleat use from a randomised trial^3^ yields estimates that are similar to the empirical estimates in our study (−0.196 (expected population impact) vs −0.235 (our empirical estimate) ice-related injuries per 1000 person-winters; see online supplemental file 1 for details.

Previous economic evaluations—one conducted alongside the impact evaluation from Gothenburg^7^ and the other a modelling study investigating hypothetical ice cleat programmes in all Swedish municipalities^9^—have reported that the economic benefits of ice cleat distribution may outweigh the costs by about 10–90 times.^7^ ^9^ In online supplemental file 1, we perform an updated cost–benefit analysis using the estimates from the present paper, finding a benefit-to-cost ratio of approximately 85 using the official estimates for benefits per pedestrian injury averted used by the Swedish Transport Administration.^23^ Using more conservative benefit estimates, the benefit-to-cost ratio decreases to approximately 10. Thus, our results support the conclusions from previous economic evaluations indicating that ice cleat distribution is likely to be cost-effective.

### Strengths and limitations

A key strength of our study is the large sample of intervention and control municipalities combined with high-quality register data on injury rates from the Swedish NPR.^11^ Using a triple differences design,^14^ we were able to control for (1) national time trends, (2) time-invariant unobserved confounders and (3) time-varying unobserved confounders that influence eligible and ineligible ages equally (eg, local weather shocks). Our data also passed several bias checks, including synthetic and negative control analyses.

Our study also has some limitations. First, our primary intention-to-treat estimate was imprecise. It seems unlikely that these programmes would be harmful considering previous research,^4 5 7 8^ but still worth noting the upper bound of the 95% CI is consistent with a small increase in risk (0.02 ice-related injuries per 1000 person-winters; table 2).

Our design also relied on younger, ineligible ages as internal controls. If there were spillover effects in terms of increased ice cleat use in these ages, our estimates might be biased towards a null effect. However, Holmberg *et al* 8 found no evidence of spillovers on younger ages in terms of ice cleat use.

Another limitation is that our injury data do not cover injuries treated in primary care, as these are not reported to the NPR. The NPR, while generally deemed to be of good quality,^11^ may also fail to capture all ice-related injuries treated in inpatient or outpatient care, the same injury may be double counted despite efforts to reduce such risks, and data quality may change over time. However, we see no reason to suspect that any of these issues would be unique to any of the comparison groups in our study.

Despite efforts to validate our programme data, our results may still be susceptible to exposure misclassification bias. We suspect that the effectiveness results, which are based on a binary exposure classification, should be less susceptible to these problems than the efficacy estimates, which also rely on correct data about the number of distributed ice cleats.

Finally, our study is observational, and we cannot rule out the possibility of residual confounding that uniquely affects ice-related injuries among older adults in municipalities with distribution programmes. Our data are also ecological, and we cannot guarantee that the patients who drive the observed reductions are those who participated in the programmes.

### Future perspectives

Important avenues for future research include studying similar interventions in other contexts, preferably with a randomised design. Longer periods of follow-up time after intervention (ours was, on average, 3.5 years) may also allow for a more precise estimation of the longevity of the impact.

As expected, our results suggest that greater reach leads to greater impact. In a previous process evaluation, we found that the strongest determinant of high reach was simply how many ice cleats the municipality purchased^6^; on average, the municipalities who participated in our survey reported that 9 out of 10 ice cleats purchased were eventually distributed. Thus, it appears that those that aim high are usually able to achieve higher reach, but more in-depth analyses of other determinants of successful implementation are still needed to enable rational decision-making about the optimal design of ice cleat distribution programmes, including the most (cost-)effective means of communication and distribution.

## Conclusion

Distributing ice cleats may be a useful and cost-effective complement to winter road maintenance for reducing the incidence of ice-related fall injuries among older adults.

## Data Availability

Data are available on reasonable request. Data may be obtained from a third party and are not publicly available. This study used data from two sources. Our programme survey data are non-sensitive and will be shared with anyone on reasonable request. The injury outcome data, although aggregated, were classified as sensitive personal data by the National Board of Health and Welfare due to the fine-grained aggregation into cells with few patients. These data must, therefore, be handled in accordance with the Swedish Ethical Review Act (SFS 2003:460) and the European Union’s General Data Protection Regulation (GDPR; 2016/679). Researchers interested in gaining access to this part of the data must first apply for ethical approval from the Swedish Ethical Review authority. With the appropriate approvals in place, the data can then be ordered directly from the National Board of Health and Welfare or by contacting the corresponding author.
