# Supplementary material for: Quasi-experimental evaluation of municipal ice cleat distribution programmes for older adults in Sweden
Source: Inj Prev. 2023 May 22;29(5):378–83. doi: 10.1136/ip-2022-044808 (PMC10579486; doi:10.1136/ip-2022-044808)
Supplement: Supplementary data [file ip-2022-044808supp001.pdf]

Eklund et al., Online Appendix

Online Supplementary Appendix

This Appendix contains additional output and analyses to support the conclusions of Eklund et al. "A quasi-experimental evaluation of municipal ice cleat distribution programs for older adults in Sweden"

CONTENTS

Supplementary figures 2

Triple differences methodology, additional details 7

Cost-benefit analysis 9

Replication code for R 10

Synthetic control analysis 14

Expected impact based on external data 16

Replication code for R 17

References 19

Eklund E, *et al. Inj Prev* 2023; 29:378–383. doi: 10.1136/ip-2022-044808

Eklund et al., Online Appendix

SUPPLEMENTARY FIGURES

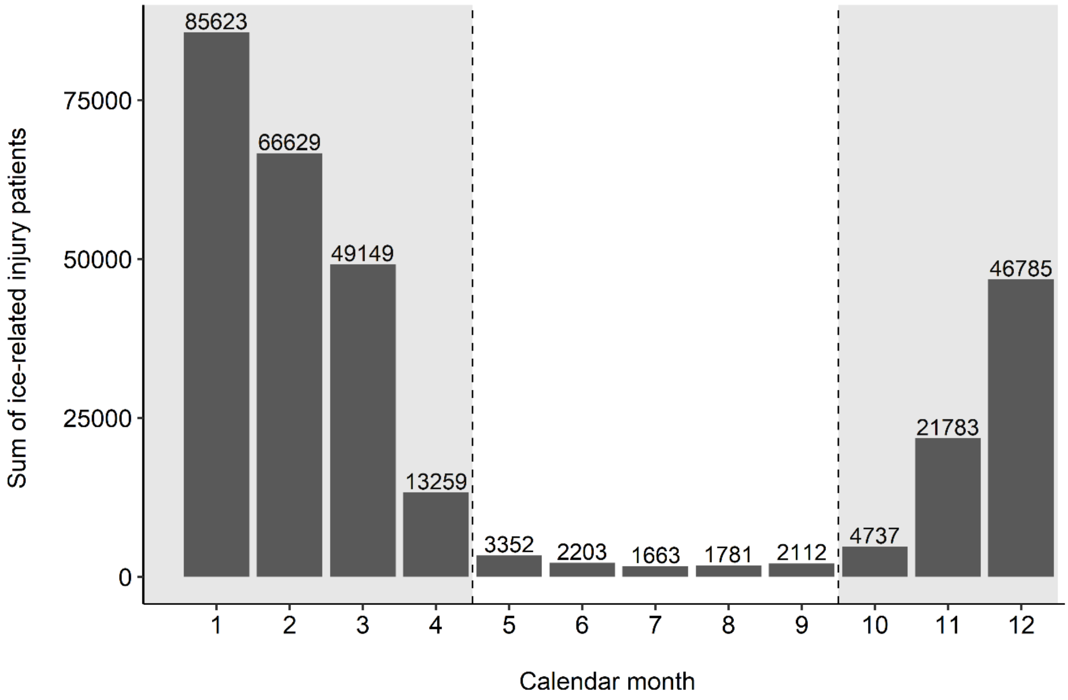

Figure S1. Total number of patients from 2001 to 2019 treated for falls due to snow and ice (ICD-10 external cause code W00) in outpatient or inpatient care according to data from the Swedish National Patient Register, by calendar month. The shaded period (October-April) is time interval used to define a winter period in our study.

Eklund E, *et al. Inj Prev* 2023; 29:378–383. doi: 10.1136/ip-2022-044808

Eklund et al., Online Appendix

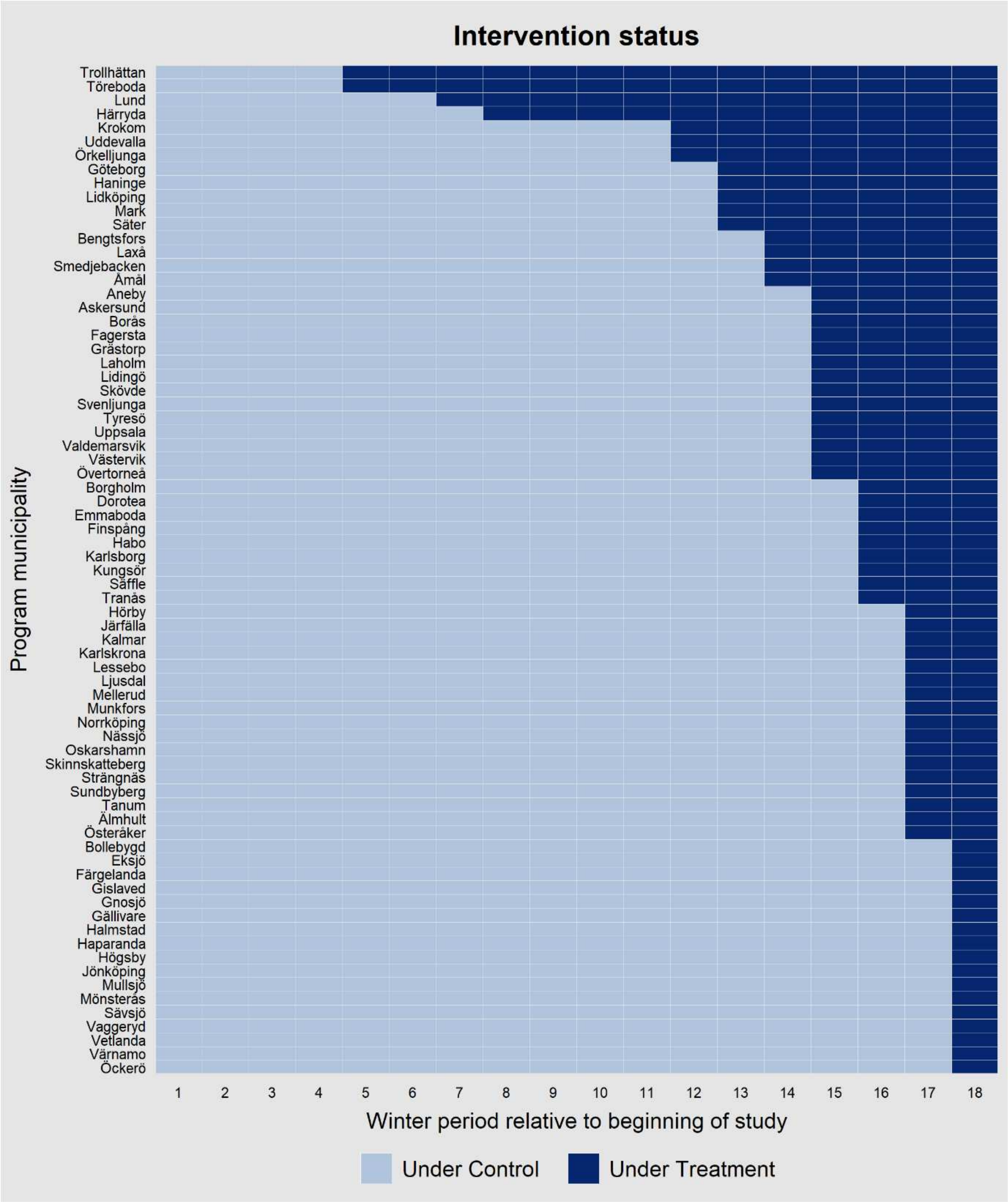

Figure S2. Timing of ice cleat distribution in each programme municipality (n = 73) by winter period relative to the beginning of the study (1 = 2001/2002; 18 = 2018/2019).

Eklund E, *et al. Inj Prev* 2023; 29:378–383. doi: 10.1136/ip-2022-044808

Eklund et al., Online Appendix

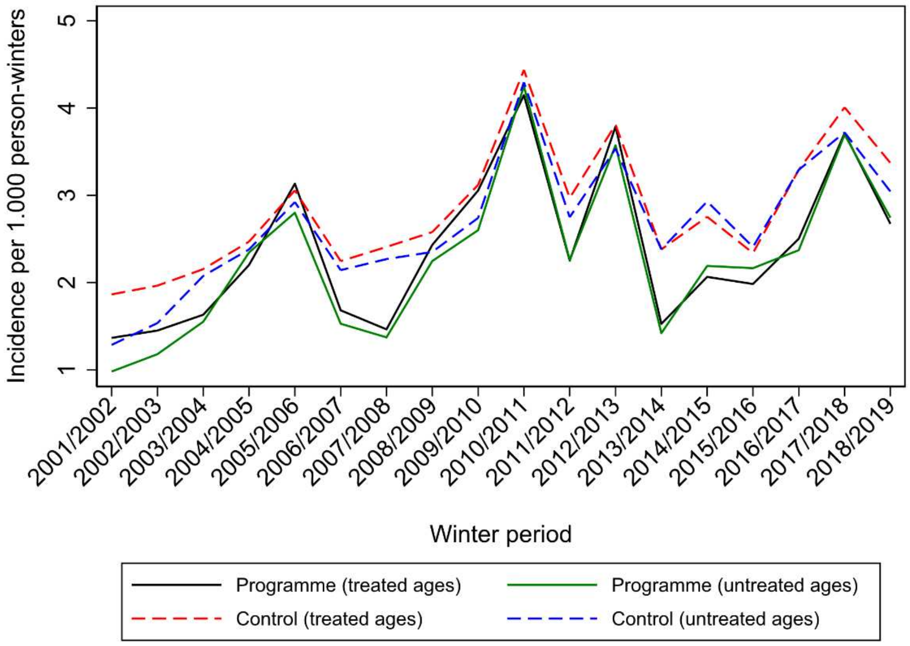

Figure S3. Trends in the incidence of ice-related fall injuries (ICD-10 external cause code W00) per intervention status and age range.

Eklund et al., Online Appendix

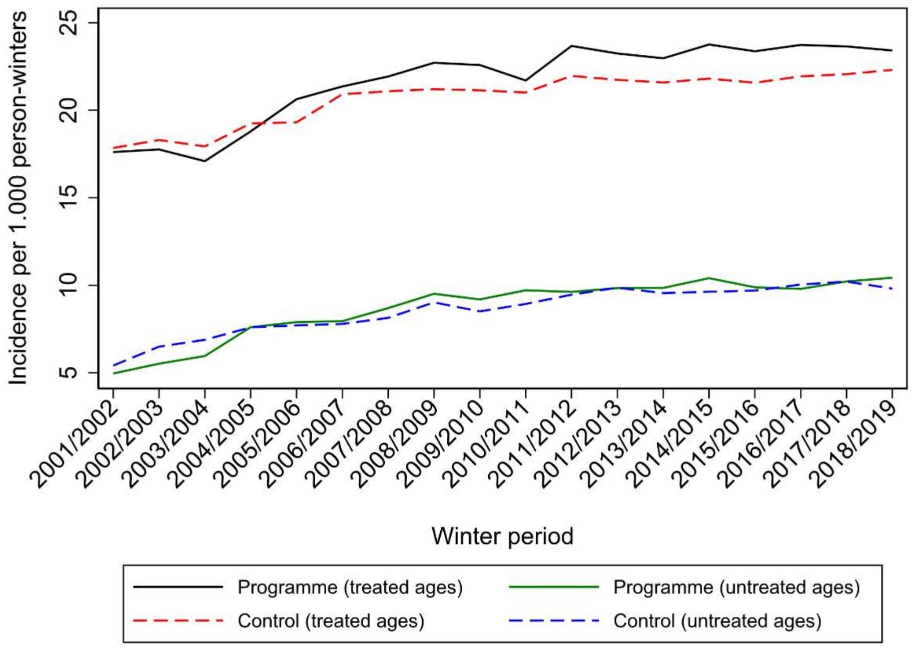

Figure S4. Trends in the incidence of fall injuries unrelated to snow and ice (ICD-10 external cause codes W01-W18) by intervention status and age range.

Eklund E, *et al. Inj Prev* 2023; 29:378–383. doi: 10.1136/ip-2022-044808

Eklund et al., Online Appendix

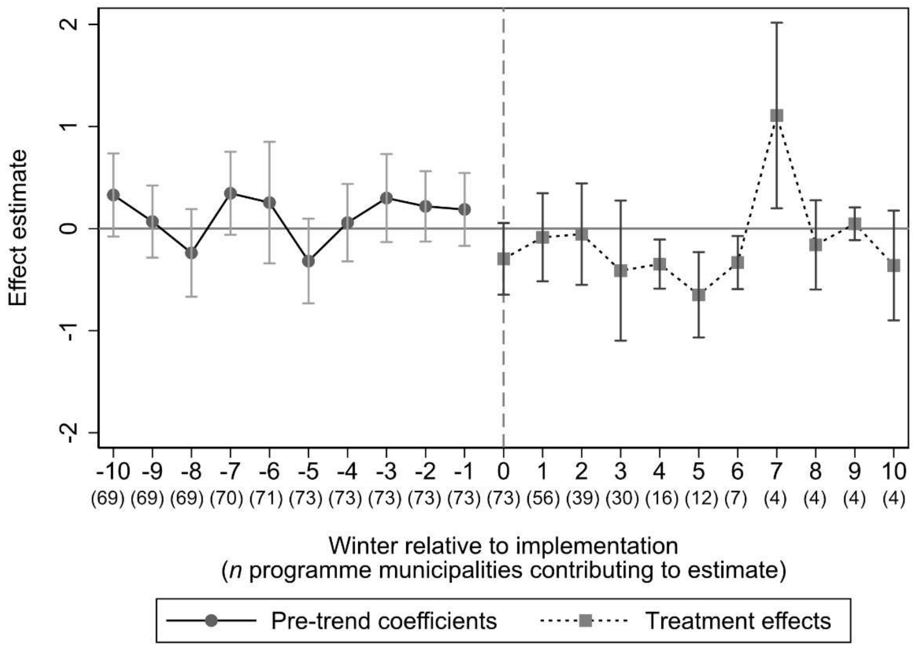

Figure S5. Event study plot showing triple differences impact estimates on ice-related fall injury incidence per 1.000 person-winters up to ten winters before and ten winters after the implementation of the ice cleat distribution programmes. Due to staggered adoption, the number of programme municipalities that contribute with data varies by time point, as detailed in parentheses in the labels on the x-axis. The pre-intervention coefficients were estimated to assess differential pre-trends (evidence of trends in pre-intervention coefficients or significant pre-intervention coefficients may be a sign of bias). The plot also includes post-intervention coefficients for reference, but we caution against interpreting variation in these given that the number of programme municipalities contributing with data drops off quickly after the first post-intervention period (variations over time may be due to the changing composition of the sample).

Eklund E, *et al. Inj Prev* 2023; 29:378–383. doi: 10.1136/ip-2022-044808

Eklund et al., Online Appendix

TRIPLE DIFFERENCES METHODOLOGY, ADDITIONAL DETAILS

This section contains additional details about the statistical methodology used to estimate the impact of ice cleat distribution programmes in our study. We used a generalized version of difference-in-differences, referred to as triple differences (or difference-in-difference-in-differences). In a regression framework, the triple differences model can be expressed as follows [1,2]:

$$Y_{igt} = \alpha_{ig} + \alpha_{it} + \alpha_{gt} + \tau D_{igt} + \epsilon_{igt} \quad (1)$$

where Y_{igt} is the outcome variable (injury incidence per 1.000 person-winters) in municipality *i*, age group *g*, and winter *t*; α_{ig} are municipality and age group-specific fixed; α_{it} are municipality and time-specific fixed effects; α_{gt} are age group and time-specific fixed effects; τ is the estimated average treatment effect on the treated; D_{igt} is an intervention dummy coded for treated observations and 0 otherwise, and ϵ_{igt} is the error term. In our case, treated observations are defined as post-intervention time points in eligible age groups within programme municipalities.

The regression-based triple differences model in Equation 1, and its standard difference-in-differences representation (without an internal control group), has recently been shown to be biased when units implement the intervention at different times (also known as staggered adoption) if treatment effects are heterogeneous [2,3]. The bias occurs due to a previously unknown problem relating to improper comparisons where early adopters (municipalities that implement early in the study period) may inadvertently serve as controls for late adopters (municipalities that implement late in the study period).

Borusyak et al. [2] recently proposed a simple way to avoid this problem using imputation. The idea builds on the potential outcomes framework, where it is typically conceptualized that each unit has two potential outcomes: one potential outcome with an ice cleat distribution program, $Y(1)_{igt}$, and one without, $Y(0)_{igt}$. The causal effect of the program for unit *i*, group *g*, and time *t*, is then given by $Y(1)_{igt} - Y(0)_{igt}$, and the average treatment effect on the treated (ATT) is given by taking

Eklund E, *et al. Inj Prev* 2023; 29:378–383. doi: 10.1136/ip-2022-044808

expectations over the post-intervention period in the treatment group, i.e., $E[Y(1)_{igt} - Y(0)_{igt}|D = 1]$, which is our target quantity.

Assuming counterfactual consistency [4], we can write $Y_{igt} = Y(1)_{igt}$ for all post-intervention observations in the treatment group. That is, we assume that in these periods and groups (where $D = 1$), the realized outcome, Y_{igt}, is the potential outcome under the treated state, $Y(1)_{igt}$. However, when $D = 1$, $Y(0)_{igt}$ is missing must be imputed to estimate the ATT.

The imputation-based estimator exploits the idea that in all other periods and groups (where $D = 0$), we observe the potential outcome under the untreated state, $Y(0)_{igt}$. The imputation estimator can be described in the following steps:

1. Subset the data to untreated observations only (i.e., when $D = 0$) and estimate a regression $Y(0)_{igt} = \alpha_{ig} + \alpha_{it} + \alpha_{gt} + \epsilon_{igt}$ to obtain estimates of all fixed effects terms in Equation 1.
2. For each treated observation (i.e., when $D = 1$), estimate the missing potential outcome by setting $\hat{Y}(0)_{igt} = \alpha_{ig} + \alpha_{it} + \alpha_{gt}$.
3. For each treated observation (i.e., when $D = 1$), estimate unit-specific treatment effects by setting $\hat{\tau}_{igt} = Y_{igt} - \hat{Y}(0)_{igt}$.
4. Estimate the ATT by taking the average of $\hat{\tau}_{igt}$ over all treated observations (i.e., when $D = 1$).

To estimate efficacy, we replace $\hat{\tau}_{igt}$ with $\frac{\hat{\tau}_{igt}}{R_i}$ in Step 4, where R_i is the number of ice cleats distributed per eligible citizen in municipality *i* (see Section 5.2 in Borusyak et al. [2]).

The imputation process solves the improper comparisons problem by only using untreated and not-yet-treated observations for model fitting. For more advanced statistical details (e.g., estimation of standard errors), please refer to reference [2].

Eklund et al., Online Appendix

COST-BENEFIT ANALYSIS

This section details a back-of-the-envelope cost-benefit analysis using the effect estimates from our study.

According to our program survey, the average incremental cost of ice cleat distribution is €3.069 (31.28 SEK, 2018) per eligible citizen. For simplicity, we assume that this investment takes place initially at the program's start. To monetize the effect on injuries, we presume a monetary benefit per averted injury of €38,576 (393,198 SEK, 2018). This number, which is derived from external data [5–7], reflects the sum of avoided societal costs excluding productivity loss (€3,592 [36,612 SEK, 2018] [5,7]) and the willingness to pay (WTP) per averted quality-adjusted life year (QALY) loss associated with a pedestrian fall injury (QALY loss per injury [5]: 0.1488[1]; WTP per QALY [6]: €235,178 [2,397,081 SEK, 2018]).

We assume that the program lasts 3.5 years, which is the average length of the post-intervention period in our empirical data. For simplicity, we assume that the effect on injury rates (0.0002351 prevented injuries per person-year according to our triple differences model) is evenly distributed over this period.

After monetizing the effect estimate and applying a discount rate of 3.5% per year for future benefits (recommended by the Swedish Transport Administration [8]), we obtain an estimated total benefit of €30.39 (309.8 SEK, 2018) per eligible citizen for the average ice cleat distribution program. Subtracting the initial investment implies a net present value of €27.32 per person (278.5 SEK, 2018; benefit-to-cost ratio: 9.9). Thus, the benefits seem to outweigh the costs from a (Swedish) societal perspective. This was also true in 94.75% of 10,000 Monte Carlo simulations accounting for sampling

[1] Our own calculation based on Table 25 in Olofsson et al [5], which contains data up to 6 months after an average pedestrian fall injury in a Swedish context. They provide a different total loss QALY estimate per person (1.387), which is based on extrapolation of the QALY loss from the year of injury to the average life expectancy in their sample. This is the official estimate currently used for economic analyses by the Swedish Transport Administration [8]. However, given the short data collection period, we take a conservative stance and assume that the health-related quality of life has returned to normal after 12 months. Re-calculation by applying the trapezoid rule [9] under this assumption which yields our conservative QALY loss estimate (0.1488). We note that using the official QALY loss estimates in our cost-benefit analysis implies a considerably larger benefit-to-cost ratio (84.65), which is very close to the model-based estimates provided in Bonander et al [7] (mean benefit-to-cost-ratio: 87), who also used the official QALY loss estimates.

Eklund E, *et al. Inj Prev* 2023; 29:378–383. doi: 10.1136/ip-2022-044808

uncertainty in the effects and program cost estimates, assuming a normal distribution for the effect and a gamma distribution for costs.

Replication code for R

```
# Seed for reproducibility
set.seed(201398)
# Avg. length of post-period in empirical study
post.period <- 3.5
# Conversion to Euro
conversion_euro = 0.09811 #Convert SEK to Euro December 31, 2018 Rate.
# QALY loss due to injury from IHE
hrq <- c(0.918,0.204,0.563,0.678,0.796,0.918) #Last point assumes return to normal at 12 months
# Calculate QALY loss
time_diff <- c(0.002739726,0.035616438,0.126027397,0.335616438,0.5) #Trapezoid calc
qaly_inj_base <- (hrq[1]+hrq[2])*time_diff[1]*0.5 +
  (hrq[2]+hrq[3])*time_diff[2]*0.5 +
  (hrq[3]+hrq[4])*time_diff[3]*0.5 +
  (hrq[4]+hrq[5])*time_diff[4]*0.5 +
  (hrq[5]+hrq[6])*time_diff[5]*0.5
qaly_healthy_base <- 0.918
# Modified benefit assuming conservative QALY loss
wtp_p_inj = 3324751 #ASEK 7.0 in 2018 SEK, official number
q_loss1 <- 1.387 #QALY loss assumed in ASEK
q_loss2 <- qaly_healthy_base-qaly_inj_base #Our conservative QALY loss assuming return to normal at 12 months
wtp_qaly <- 3324751/1.387
wtp_modified <- q_loss2*wtp_qaly
# Healthcare costs (subtracting production loss) from IHE report
hc_cost <- 36612
# Average treatment effect estimates
effect <- (-.2350829/1000)
effect_se <- (((.0151396/1000)-(-.4853054/1000))/3.92)
dist_prevented <- -rnorm(10000,effect,effect_se) #Flip sign to get injuries prevented
```

Eklund et al., Online Appendix

```
benefit_per_prevented <- (wtp_modified+hc_cost)*conversion_euro

benefit <- (-effect)*benefit_per_prevented

dist_benefit <- dist_prevented*benefit_per_prevented

# Average cost per person

cost_mean <- 31.28006*conversion_euro #From our survey data

cost_se <- 3.860791*conversion_euro #From our survey data

cost_alpha <- (cost_mean/cost_se)^2

cost_beta <- (cost_se^2)/cost_mean

cost <- cost_mean

dist_cost <- rgamma(10000,shape=cost_alpha,scale=cost_beta)

# Discount rate

d <- 0.035

# Calculate base case results

npv.list=cost.list=benefit.list=list()

for (t in 1:4) {

   if (t == 1) {

   npv.list[[t]] <- (benefit-cost)

   benefit.list[[t]] <- benefit

   cost.list[[t]] <- cost

  }

  else {

   npv.list[[t]] <- (benefit/((1+d)^(t-1)))

   benefit.list[[t]] <- (benefit/((1+d)^(t-1)))

   cost.list[[t]] <- 0

  }

  if (t == 4) { #Half benefit final year to account for 3.5 yrs of post-period data

   npv.list[[t]] <- npv.list[[t]]*0.5

   benefit.list[[t]] <- benefit.list[[t]]*0.5

  }

}

npv.res <- sum(do.call("rbind",npv.list))

benefit.res <- sum(do.call("rbind",benefit.list))

cost.res <- sum(do.call("rbind",cost.list))

bca.res <- benefit.res/cost.res
```

```
base.res <- c(benefit.res,cost.res,npv.res,bca.res)
# Probabilistic sensitivity analysis (PSA) function
sim.fun <- function(b,c) {
 npv.list=cost.list=benefit.list=list()
 for (t in 1:4) {
  if (t == 1) {
   npv.list[[t]] <- (b-c)
   benefit.list[[t]] <- b
   cost.list[[t]] <- c
  }
  else {
   npv.list[[t]] <- (b/((1+d)^(t-1)))
   benefit.list[[t]] <- (b/((1+d)^(t-1)))
   cost.list[[t]] <- 0
  }
    if (t == 4) { #Half benefit final year to account for 3.5 yrs of post-period data
   npv.list[[t]] <- npv.list[[t]]*0.5
   benefit.list[[t]] <- benefit.list[[t]]*0.5
  }
 }
 npv.res <- sum(do.call("rbind",npv.list))
 benefit.res <- sum(do.call("rbind",benefit.list))
 cost.res <- sum(do.call("rbind",cost.list))
 bca.res <- benefit.res/cost.res
 sim.res <- data.frame(benefit.res,cost.res,npv.res,bca.res)
 return(sim.res)
}
# Loop the PSA function
sim.list <- list()
for (i in 1:10000) {
 sim.list[[i]] <- sim.fun(b=dist_benefit[[i]],c=dist_cost[[i]])
}
sim.df <- do.call("rbind",sim.list)
benefit.lower <- quantile(sim.df[,1],0.025)
```

```
benefit.upper <- quantile(sim.df[,1],0.975)

cost.lower <- quantile(sim.df[,2],0.025)

cost.upper <- quantile(sim.df[,2],0.975)

npv.lower <- quantile(sim.df[,3],0.025)

npv.upper <- quantile(sim.df[,3],0.975)

bca.lower <- quantile(sim.df[,4],0.025)

bca.upper <- quantile(sim.df[,4],0.975)

prob.costbenefit <- mean(sim.df$npv.res>0)
```

SYNTHETIC CONTROL ANALYSIS

This section details a sensitivity analysis to assess if our main estimates are sensitive to non-parallel trends by running a synthetic control analysis. Synthetic controls are a generalization of the difference-in-differences framework that can handle situations where pre-intervention trends diverge across units.

To implement the method, we applied the Bayesian dynamic multilevel latent factor model framework proposed by Pang et al. [10]. For our purposes, the benefits of this framework are three-fold: (i) it provides easily interpretable credible intervals for the effect estimates, (ii) it accepts outcomes among younger ages as time-varying covariates with municipality-specific coefficients, (iii) it allows for coefficient shrinkage on time-varying covariates to avoid overfitting, which is important when including noisy outcomes as covariates. The method helps handle situations with non-parallel trends in addition to estimating municipality and time fixed effects. In practice, this is done by subsetting the data to not-yet-treated observations and estimating latent time-varying factors and constant municipality-specific factor loadings; municipalities with similar factor loadings share similar trends. The observed counterfactual outcomes are then imputed based on the model.

We used the *bpCausal* package for R to run the analysis [10]. The package uses Markov Chain Monte Carlo (MCMC) algorithm to estimate parameters and perform model selection. Our model included the incidence of ice-related fall injuries per 1.000 person-winters in the treated age range as the outcome variable; a post-intervention treatment dummy, coded as one after the intervention in treated municipalities and zero otherwise; and the incidence of ice-related fall injuries per 1.000 person-winters in the negative control ages as a time-varying covariate. Following Pang et al. [10], we allowed for up to 10 latent factors. We also allowed the time-varying covariate to have a common (constant) fixed effect, municipality-level random effects, and time-level random effects. Coefficient shrinkage was used on all effects and on the factor loadings to assist with model selection and avoid overfitting. Priors on the shrinkage were set to Gamma(0.001, 0.001), as recommended by Pang et al. [10]. We performed 50,000 MCMC runs, discarding the first 5,000 runs as a burn-in period.

Eklund et al., Online Appendix

The results are presented in Figure S5. We note that the pre-trends are consistently close to zero, implying that the method successfully handled non-parallel trends in the pre-intervention period. The average post-intervention estimate is -0.220 (95% credible interval: -0.445, 0.004) ice-related fall injuries per 1,000 person-winters, which is very similar to our primary triple differenecs estimate (-0.235 [95% confidence interval: -0.485, 0.015]). Thus, our initial estimates appear robust to non-parallel pre-trends.

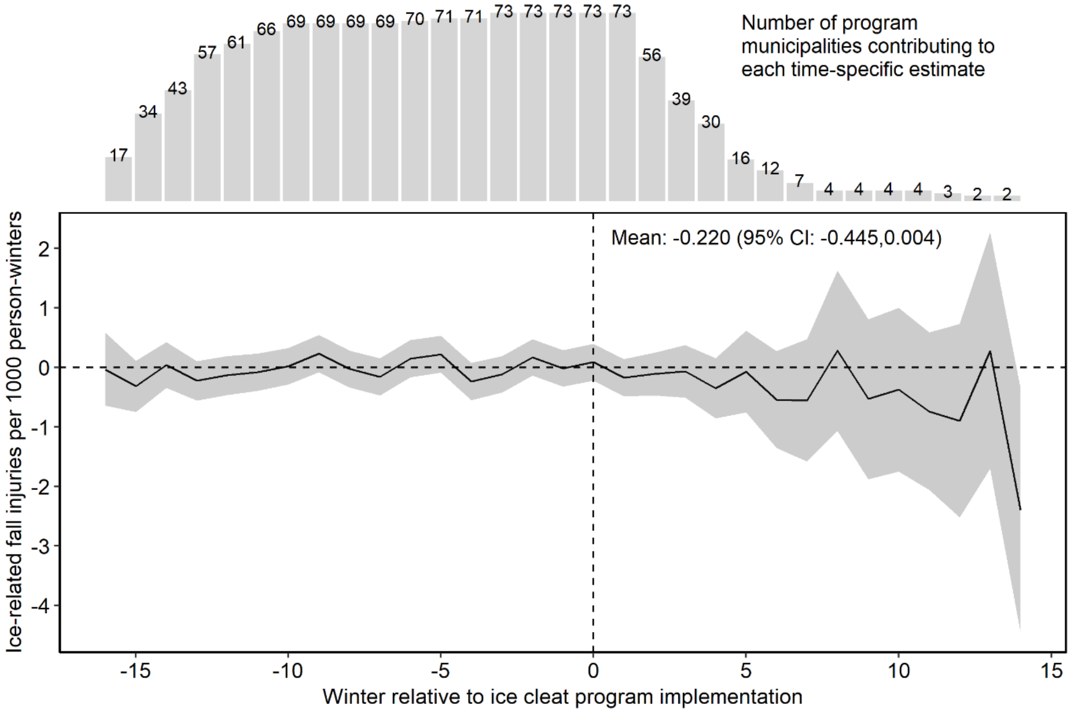

Figure S6. Estimated time-varying effects (incl. pre-trends) relative to the implementation of ice cleat programs using Bayesian synthetic controls. The number of program municipalities contributing to each time point varies, as shown in the bar chart above the plot, due to time-varying adoption dates. The mean estimate is the average of all unit- and time-specific post-intervention effect estimates (early post-intervention years contribute the most to this average due to the higher number of program municipalities contributing with data in those periods).

Eklund et al., Online Appendix

EXPECTED IMPACT BASED ON EXTERNAL DATA

This section details a calculation of the expected impact of ice cleat distribution programs based on external data sources. We use this methodology to assess the plausibility of the estimates obtained in our main analysis.

We used data from two external sources to conduct a population impact analysis [11] to quantify the expected average impact in the 73 program municipalities included in our study. The first source is a randomized controlled trial evaluating the effects of ice cleat use among older adults in the US [12]. The other is an observational study investigating the impact of ice cleat distribution programs in Sweden on ice cleat use [13].

We applied the population impact analysis formula detailed in Heller et al. [11] to estimate the expected number of ice-related injuries prevented per 1.000 person-winters. The estimate is given by:

$$y_0\left(\frac{\Delta(1/RR-1)}{1+\Delta(1/RR-1)}\right),$$

where y_0 is the mean incidence rate per 1000 person-winters before implementation (obtained from our data); Δ is the average causal effect of ice cleat distribution programs on ice cleat use, expressed as a probability difference (0.075; obtained from [13]); and RR is the average causal risk ratio associated with ice cleat use (0.45; obtained from [12]). We performed 10,000 Monte Carlo simulations to assess uncertainty in the expected impact.

The results are reported in Table S1. According to the impact analysis, we can expect an effect of -0.1959 ice-related injuries per 1,000 person-winters with a 0.075 probability increase in ice cleat use and a causal risk ratio of 0.45. The expected impact estimate is close to the empirical estimate from the present study (-0.2350), suggesting that the empirical estimate is within a plausible range.

Eklund E, *et al. Inj Prev* 2023; 29:378–383. doi: 10.1136/ip-2022-044808

Eklund et al., Online Appendix

Table S1. Comparison of the empirical estimates of the effects of ice cleat distribution programs on ice-related injury rates among older adults in Sweden from the present study to estimates based on population impact analysis using external data.

	Mean	SE	Lower 95%	Upper 95%
**Input data**				
Change in ice cleat use (probability difference, Δ)	0.075	0.0169	0.042	0.108
Risk reduction associated with ice cleat use (RR)	0.45		0.23	0.85
Baseline injury rate per 1,000 person-winters (y_0)	2.326	0.055	2.217	2.434
**Expected effect based on external data**				
Effect per 1.000 person-winters (rate difference)	-0.1959	0.1189	-0.4845	-0.0245
**Empirical estimates from the present study**				
Effect per 1.000 person-winters (rate difference)	-0.2350	0.1277	-0.4853	0.0151

Notes: SE = Standard error. 95% confidence intervals for expected effects were estimated using Monte Carlo simulations with 10,000 replicates, assuming a normal distribution on all parameters except the relative risk, RR, which was simulated assuming a log-normal distribution.

Replication code for R

```
## Set seed for reproducibility
set.seed(102398123)
## Define input parameters
# RR (McKiernan)
lnRR = log(0.45)
seRR = (log(0.85)-log(0.23))/3.92
# Baseline rate (our data)
baseline_rate <- 2.325701
baseline_rate_se <- .0552909
# Change in use (Holmberg et al)
change_in_use <- .0752676
change_in_use_se <- .0168791
# Perform impact analysis, base case
impact_derived <- baseline_rate*((change_in_use*(1/exp(lnRR)-1))/(1+change_in_use*(1/exp(lnRR)-1)))
# Simulate uncertainty
sim_change <- rnorm(10000,change_in_use,change_in_use_se)
```


Eklund E, *et al. Inj Prev* 2023; 29:378–383. doi: 10.1136/ip-2022-044808

```
sim_logrr <- rnorm(10000,lnRR,seRR)

sim_rate <- rnorm(10000,baseline_rate,baseline_rate_se)

sim_impacts <- sim_rate * ((sim_change*(1/exp(sim_logrr)-1)) / (1+sim_change*(1/exp(sim_logrr)-1)))

quantile(sim_impacts,c(0.025,0.975))
```

REFERENCES

1 Olden A, Møen J. The triple difference estimator. *Econom J* 2022;:utac010. doi:10.1093/ectj/utac010

2 Borusyak K, Jaravel X, Spiess J. Revisiting Event Study Designs: Robust and Efficient Estimation. *ArXiv210812419 Econ* Published Online First: 27 August 2021.http://arxiv.org/abs/2108.12419 (accessed 22 Dec 2021).

3 Goodman-Bacon A. Difference-in-differences with variation in treatment timing. *J Econom* 2021;**225**:254–77. doi:10.1016/j.jeconom.2021.03.014

4 Hernán MA. Does water kill? A call for less casual causal inferences. *Ann Epidemiol* 2016;**26**:674–80. doi:10.1016/j.annepidem.2016.08.016

5 Olofsson S, Gralén K, Macheridis K, *et al.* Personskadekostnader och livskvalitetsförlust till följd av vägtrafikolyckor och fotgängarolyckor singel. Sammanfattning av resultat. Lund: : Lund University 2016. https://ihe.se/publicering/sammanfattning-personskadekostnader-och-livskvalitetsforlust/ (accessed 18 Feb 2021).

6 Olofsson S, Persson U, Hultkrantz L, *et al.* Betalningsviljan för att minska risken för icke-dödliga och dödliga skador i samband med vägtrafikolyckor – en studie med kedje-ansats. Lund: : Lund University 2016. https://ihe.se/publicering/betalningsviljan-att-minska-risken-icke-dodliga-och-dodliga-skador-samband-med-vagtrafikolyckor-en-studie-med-kedje-ansats/ (accessed 18 Feb 2021).

7 Bonander C, Holmberg R, Gustavsson J, *et al.* Model-based economic evaluation of ice cleat distribution programmes for the prevention of outdoor falls among adults from a Swedish societal perspective. *Inj Prev* Published Online First: 27 June 2021. doi:10.1136/injuryprev-2021-044203

8 The Swedish Transport Administration. Analysmetod och samhällsekonomiska kalkylvärden för transportsektorn. 2020. https://www.trafikverket.se/contentassets/4b1c1005597d47bda386d81dd3444b24/asek-7-hela-rapporten_210129.pdf (accessed 18 Feb 2021).

9 Brazier J, Ratcliffe J, Saloman J, *et al. Measuring and Valuing Health Benefits for Economic Evaluation*. Oxford University Press 2017.

10 Pang X, Liu L, Xu Y. A Bayesian Alternative to Synthetic Control for Comparative Case Studies. *Polit Anal* 2021;:1–20. doi:10.1017/pan.2021.22

11 Verma A, Torun P, Harris E, *et al.* Population Impact Analysis: a framework for assessing the population impact of a risk or intervention. *J Public Health Oxf Engl* 2012;**34**:83–9. doi:10.1093/pubmed/fdr026

12 McKiernan FE. A simple gait-stabilizing device reduces outdoor falls and nonserious injurious falls in fall-prone older people during the winter. *J Am Geriatr Soc* 2005;**53**:943–7. doi:10.1111/j.1532-5415.2005.53302.x

13 Holmberg R, Gustavsson J, Svensson M, *et al.* Ice cleat distribution programmes and ice cleat use among older adults: repeated cross-sectional evidence from 63 municipal interventions in Sweden. *Inj Prev* Published Online First: 2 August 2022. doi:10.1136/ip-2022-044681
